# Association Between Patient Sex and Familial Hypercholesterolemia and Long-Term Cardiovascular Risk Factor Management 5 Years After Acute Coronary Syndrome

**DOI:** 10.1161/CIRCOUTCOMES.123.010790

**Published:** 2024-06-20

**Authors:** Kristina Krasieva, Baris Gencer, Isabella Locatelli, David Carballo, Olivier Muller, Stéphane Fournier, Christian M. Matter, Lorenz Räber, Nicolas Rodondi, François Mach, David Nanchen

**Affiliations:** Center for Primary Care and Public Health (Unisanté), University of Lausanne, Switzerland (K.K., I.L., D.N.).; Service of Cardiology, Geneva University Hospital, Switzerland (B.G., D.C., F.M.).; Institute of Primary Health Care (BIHAM), University of Bern, Switzerland (B.G., N.R.).; Service of Cardiology, Lausanne University Hospital, Switzerland (B.G., O.M., S.F.).; University Heart Center, Department of Cardiology, Zurich University Hospital, and Center of Translational and Experimental Cardiology, Department of Cardiology, Zurich University Hospital and University of Zurich, Switzerland (C.M.M.).; Department of Cardiology, Inselspital, Bern University Hospital, University of Bern, Switzerland (L.R.).; Department of General Internal Medicine, Inselspital, Bern University Hospital, Switzerland (N.R.).

**Keywords:** acute coronary syndrome, hyperlipoproteinemia type II, lipids, lipoproteins, LDL, risk factors

## Abstract

**BACKGROUND:**

Long-term control of cardiovascular risk factors after acute coronary syndrome (ACS) is the cornerstone for preventing recurrence. We investigated the extent of cardiovascular risk factor management in males and females with and without familial hypercholesterolemia (FH) 5 years after ACS.

**METHODS:**

We studied patients hospitalized for ACS between 2009 and 2017 in a Swiss multicenter prospective cohort study. FH was defined based on clinical criteria from the Dutch Lipid Clinic Network and Simon Broome definitions. Five years post-ACS, we assessed low-density lipoprotein-cholesterol (LDL-c) levels, lipid-lowering therapy (LLT), and other cardiovascular risk factors, comparing males to females with and without FH using generalized estimating equations.

**RESULTS:**

A total of 3139 patients were included; mean age was 61.4 years (SD, 12.1), 620 (19.8%) were female, and 747 (23.5%) had possible FH. Compared with males at 5-years post-ACS, females were more likely to not use statins (odds ratio, 1.61 [95% CI, 1.28–2.03]) and less likely to have combination LLT (odds ratio, 0.72 [95% CI, 0.55–0.93]), without difference between patients with FH and without FH. Females in both FH and non-FH groups less frequently reached LDL-c values ≤1.8 mmol/L (odds ratio, 0.78 [95% CI, 0.78–0.93]). Overall, patients with FH were more frequently on high-dose statins compared with patients without FH (51.0% versus 42.9%; *P*=0.001) and presented more frequently with a combination of 2 or more LLT compared with patients without FH (33.8% versus 17.7%; *P*<0.001), but less frequently reached LDL-c targets of ≤1.8 mmol/L (33.5% versus 44.3%; *P*<0.001) or ≤2.6 mmol/L (70.2% versus 78.1%; *P*=0.001).

**CONCLUSIONS:**

Five years after ACS, females had less intensive LLT and were less likely to reach target LDL-c levels than males, regardless of FH status. Males and females with FH had less optimal control of LDL-c despite more frequently taking high-dose statins or combination LLT compared with patients without FH. Long-term management of patients with ACS and FH, especially females, warrants optimization.

**WHAT IS KNOWN**

- Females are less likely to have intensive lipid-lowering treatment than men, such as high-dose statins or a combination of different lipid-lowering therapies.
- Most patients with familial hypercholesterolemia (FH) failed to reach recommended low-density lipoprotein-cholesterol levels.

**WHAT THIS STUDY ADDS**

- Five years after acute coronary syndrome, sex disparities are present in FH management, with females less likely to take statins or have a combination of lipid-lowering therapies.
- Females reached less frequently target low-density lipoprotein-cholesterol values than males in the 5 years after acute coronary syndrome, independent of the presence of FH.
- Patients with clinical FH have more intensive lipid-lowering treatment than patients without FH. Despite this, patients with FH are less likely to reach guidelines-recommended low-density lipoprotein-cholesterol levels than those without FH.

Sex differences in cardiovascular therapy among patients with coronary artery disease (CAD) have been reported in both hospital and outpatient settings and among different groups of patients.^1,2^ Worldwide, females with familial hypercholesterolemia (FH) were less likely to be taking high-intensity statins or a combination of lipid-lowering therapies (LLTs) than males.^3^ Patients with FH are a subpopulation that is at increased cardiovascular risk driven by life-long exposure to high levels of low-density lipoprotein-cholesterol (LDL-c). It is the long-term lowering of LDL-c that is associated with lower cardiovascular risk compared with short-term lowering.^4^ However, despite high-intensity LLT, it is more difficult for patients with FH to reach LDL-c target values.^5–8^ Some studies have also shown that it is even more difficult for patients with FH and atherosclerotic cardiovascular disease (ASCVD) to reach these targets.^9–12^ In addition, females were less likely to be taking any LLT and were less likely to reach LDL-c goal values than males.^3,13–15^ Despite similar lipid profiles, females with FH were also less likely to receive high-dose statins compared with males with FH in primary and secondary prevention.^10,16^ One year after acute coronary syndrome (ACS) in Switzerland, we reported that nearly all patients with FH had failed to reach the 2016 European Society of Cardiology (ESC) guidelines-recommended LDL-c target of <1.8 mmol/L and that for more than a fourth of patients, statin dosage was not optimized.^17^ Similar findings have been reported in other countries.^18,19^

To improve lipid management after ACS, recent 2021 ESC guidelines recommend using a combination of LLTs, such as adding ezetimibe and PCSK9 (proprotein convertase subtilisin-kexin type 9) inhibitors to statins when target values are difficult to achieve. Among patients with FH, it has been reported that combination therapy is associated with a greater reduction of LDL-c levels and higher rates of attainment of LDL-c goals than with monotherapy.^3^ These studies did not evaluate long-term outcomes post-ACS, and data remains scarce about lipid and cardiovascular risk factor management in the long term after ACS in males and females with FH. A better assessment of long-term quality of care is important because a report with a follow-up of as long as 5 years in patients with FH has shown that early and consistent LLT decreases the risk of incident ASCVD compared with those without LLT.^20^ Therefore, we aimed to investigate the association between patient sex and LDL-c, LLT, and other cardiovascular risk factor management 5 years after ACS in those with and without FH.

## METHODS

### Study Design

We performed an analysis of the SPUM-ACS (Special Program University Medicine-Acute Coronary Syndrome) cohort study database. SPUM-ACS is a large prospective multicenter cohort study of patients hospitalized with ACS in 4 university centers in Switzerland.^21^ The data that support the findings of this study are available from the corresponding author upon reasonable request. Data collection about medication use and a blood draw to measure cholesterol levels and other parameters were completed during patient visits, which took place at 4 time points: baseline, discharge, 1 year, and 5 years post-ACS. Clinical management data at 5-year follow-up visits was available for a sample of patients because they were ineligible due to study design or they did not respond to invitations to participate in the 5-year follow-up (cf. Figure S1).

### Study Population

Patients from the SPUM-ACS study were enrolled between 2007 and 2017. Patients who died during the study or who did not have follow-up data available at 5 years post-ACS were excluded.

### Diagnosis of FH

We used the Dutch Lipid Clinic Network (DLCN) score as well as the Simon Broome criteria to define the clinical FH of patients at baseline. The DLCN score is based on age, personal and family history of premature cardiovascular disease, and LDL-c levels at baseline. For patients not receiving lipid-lowering treatment at baseline, LDL-c levels without treatment were estimated based on the methods explained by Besseling et al.^22^ The DLCN score is used by many guidelines to diagnose clinical FH due to genetic tests being costly.^23–25^ The methods used to define FH based on DLCN and Simon Broome scores are described in our previous analysis at 1-year post-ACS.^17^ We further use a combination of definitions to define the presence or absence of FH: all FH definitions (DLCN, ≥3 points and Simon Broome criteria combined), possible FH (DLCN, 3–5 points), probable/definite FH (DLCN, >5 points), and possible FH based on the Simon Broome criteria.

### Outcomes

The attainment of LDL-c target levels for our study sample, comprising only very high-risk patients, was evaluated according to 4 criteria: (1) the 2019 ESC-recommended LDL-c target of ≤1.4 mmol/L for very high-risk patients; (2) the 2016 ESC-recommended target LDL of ≤1.8 mmol/L for very high-risk patients; (3) 2016 ESC-recommended target LDL of ≤2.6 mmol/L for high-risk patients; and (4) the 2016 and 2019 ESC recommendations of a decrease of at least 50% from baseline LDL-c levels.^4,26^

LLT included statins, ezetimibe, fibrates, niacin, or PCSK9 inhibitors. High-dose statin was defined as atorvastatin ≥40 mg/d or rosuvastatin ≥20 mg/d. Monotherapy was defined as the use of solely 1 LLT. Combination therapy was defined as the combination of any of the LLTs mentioned above. Management of other cardiovascular risk factors included blood pressure (BP) control, defined as a systolic BP <140 mm Hg and diastolic BP <90 mm Hg at the patients’ follow-up visit, glycated hemoglobin (HbA1c) <7% in patients with diabetes at baseline, taking aspirin and antihypertensive medication, defined as angiotensin-converting enzyme inhibitors, angiotensin II-receptor inhibitors, beta-blockers, calcium channel blockers or diuretics, smoking cessation in those who were smokers at baseline, weight loss of at least 5% in patients who were overweight or obese at baseline (overweight defined as a body mass index 25–30 kg/m^2^, obesity defined as a body mass index >30 kg/m^2^), and alcohol consumption reduction, defined as a consumption of ≤14 units of alcohol per week in patients who had an at-risk consumption at baseline, defined as >14 units per week.

### Covariables

A family history of premature CAD was defined as a history of myocardial infarction or percutaneous coronary intervention in first-degree male family members before 55 years of age and first-degree female family members before 60 years of age.

### Statistical Analyses

χ^2^ tests were used to compare binary characteristics between males and females with and without FH (all definitions combined). Continuous variables were compared using Student *t* tests as they showed an approximately normal distribution. We also performed analyses on the whole study sample (both sexes combined) with stratification based on the different definitions of FH (DLCN, Simon Broome criteria). Four generalized estimating equation models with autoregressive correlation structure were estimated to determine the impact of FH, sex, and time on (1) the probability of not taking a statin, (2) the probability of having a combination therapy, (3) the probability of having LDL-c ≤1.8 mmol/L, and (4) the probability of having LDL-c ≤2.6 mmol/L. All pairwise and 3-way interactions were tested between the 3 predictors (FH, sex, and time), retaining only significant interactions in the model (*P*<0.10). Since time is a variable with 3 categories (discharge, 1-year, and 5-years) and therefore coded with 2 binary variables, the interactions sex/time and FH/time were retained if at least one of the 2 interactions involved in each was significant (eg, FH with 1 year or FH with 5 years). Sankey charts were also added to help visualize this evolution in patients with and without FH. Stata16 software (StataCorp, College Station, TX) was used for all the statistical analyses except the Sankey charts and generalized estimating equation models, which were done with software R (R Core Team, 2022; https://www.R-project.org/). The Sankey charts were made using the package “ggsankey” (https://github.com/davidsjoberg/ggsankey.git).

### Ethics Committee

The SPUM-ACS study was approved by the medical ethics committee of each center (Lausanne, Geneva, Bern, and Zurich) at the beginning of the data collection. All participants gave written informed consent to participate in the study.

## RESULTS

At the time of analysis, a total of 6359 patients were in the SPUM-ACS cohort, and 5287 were eligible for analysis (cf. Figure S1). Of these, 59.4% participated in their 5-year follow-up visit. Therefore, our final sample for analysis consisted of 3139 patients with 5-year follow-up information on clinical management. Although the patients who did not participate in the 5-year follow-up were older and had a lower education level than those who did, the proportion of females was similar between the 2 groups, as reported in Table S1. The mean age of the 3139 participants at baseline was 61.4 years, and 620 (19.8%) were female. Table 1 shows the baseline characteristics of the participants stratified by sex and FH status. The same data are available stratified only by FH status with various definitions of FH in Table S2. In both groups, males were younger than females by 6 and 7 years at the time of hospitalization (Table 1). Overall, patients with FH were on average >10 years younger than those without FH at hospital admission and less frequently suffered from comorbidities, such as hypertension (56.4% versus 38.0%; *P*<0.001), diabetes (17.7% versus 10.7%; *P*<0.001), or preexisting cardiovascular disease (24.3% versus 15.2%; *P*<0.001), than patients without FH (Table S2).

**Table 1.**
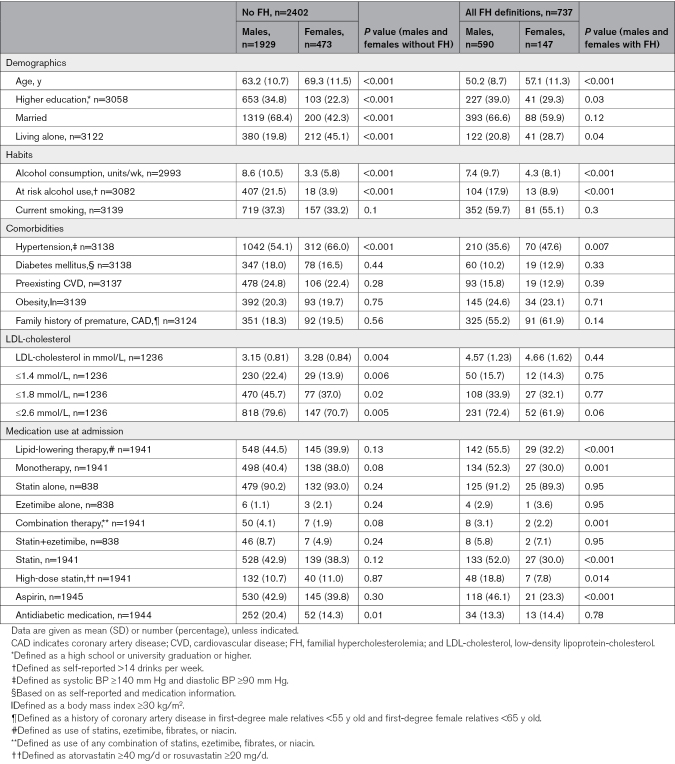
Characteristics of Study Participants at the Time of Acute Coronary Syndrome (ACS), Stratified by Sex and FH Status (n=3139)

Table 2 reports LDL-c management and type of LLT use in males and females with and without FH at 5-year follow-up post-ACS. Females more frequently presented with a higher LDL-c than males in both FH and non-FH groups (no FH: 2.26 mmol/L [SD, 0.96] versus 2.02 mmol/L [SD, 0.85]; *P*<0.001; FH: 2.55 mmol/L [SD, 1.19] versus 2.24 mmol/L [SD, 0.94]];, *P*=0.01) and were less likely to attain LDL-c targets in the no FH group (LDL-c ≤2.6 mmol/L: 70.7% versus 79.6%; *P*=0.005; Table 2). In the non-FH group, females were less frequently taking any type of LLT or having a combination LLT than males (84.0% versus 88.3%, *P*=0.04, and 14.7% versus 18.4%, *P*=0.06, respectively). A similar trend is present in the FH group without reaching statistical significance. Overall, patients with FH less frequently reach target LDL-c levels compared with patients without FH. 51.0% of patients with FH were still on high-dose statins as prescribed at hospital discharge, and 33.8% were on a combination therapy 5 years post-ACS, as compared with 42.9% (*P*=0.001) and 17.7% (*P*<0.001) of patients without FH, respectively (Table S3; Figures S2, S3, and S4). Table 3 and Table S4 report other cardiovascular risk factor management 5 years after ACS. There was no difference in the achievement of recommended targets for BP, HbA1c, smoking cessation, or alcohol use between males and females in both FH and non-FH groups. Tables S5 and S6 compare LLT use and statin intensity between males and females with and without FH and show results in terms of percentages and *P* values.

**Table 2.**
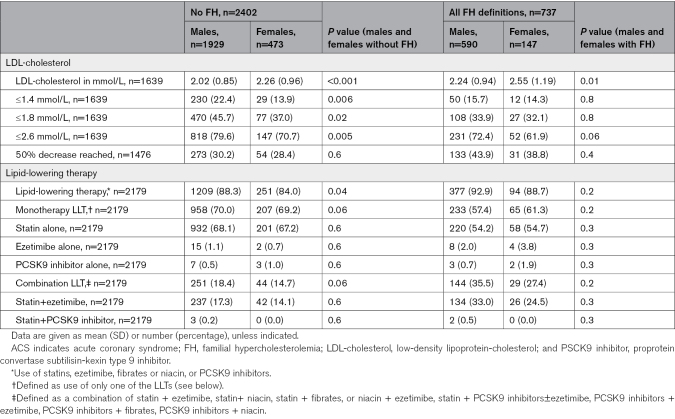
LDL-Cholesterol Goal Attainment and Lipid-Lowering Therapy in Patients 5 Years After ACS Stratified by Sex and FH Status (n=3139)

**Table 3.**
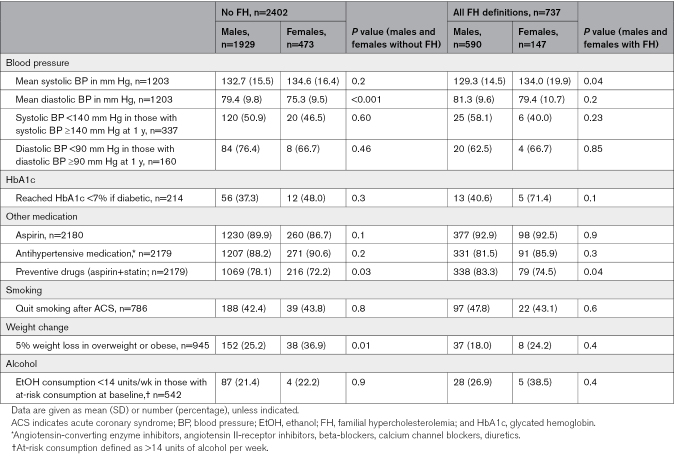
Cardiovascular Risk Factor Management in Patients 5 Years After ACS, Stratified by Sex and FH Status (n=3139)

**Figure 1.**
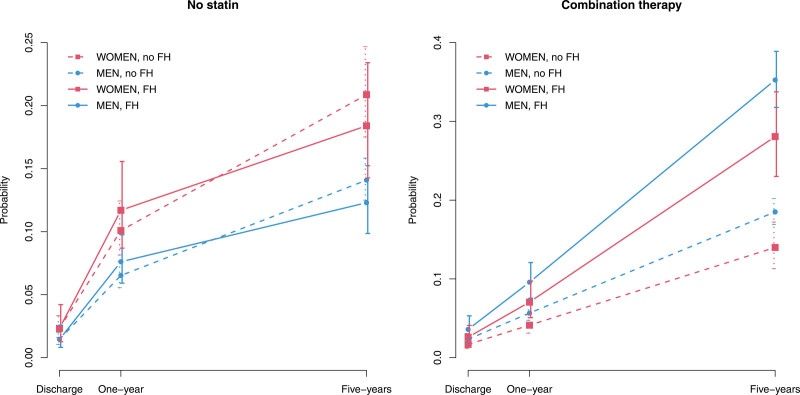
Progression of LLT and statin use from discharge to 5 years post-ACS for males and females with and without FH* estimated via generalized estimating equation (GEE) models (n=3139). *All definitions of FH used (DLCN possible and probable FH and Simon Broome register). ACS indicates acute coronary syndrome; FH, familial hypercholesterolemia; and LLT, lipid-lowering therapy.

Figure 1 and Table S7 report the progression of statin and combination therapy use over 5 years by sex and presence of FH estimated via generalized estimating equation models. Being female corresponded to a greater probability of not having statins at discharge (odds ratio [OR], 1.6 [95% CI, 1.3–2.0]), while being FH was not significantly associated with this probability (OR, 1.2 [95% CI, 0.9–1.6]). The probability of not taking statins increased significantly between discharge and 1 year, with no difference between FH and non-FH groups (non-FH: OR, 4.7 [95% CI, 3.4–6.6]; FH: OR, 5.6 [95% CI, 3.1–10.2]; *P* value of difference=0.630). The increase was also significant between 1 and 5 years, and, in this case, it was significantly greater for non-FH at the 10% significance level (no FH: OR, 2.4 [95% CI, 2.0–2.8]; FH: OR, 1.7 [95% CI, 1.2–2.4]; *P* value of the difference=0.097). No interaction between sex and time or between sex and FH was found to be significant, thus it was not retained in the model. Similarly, being female corresponded to a smaller probability of having a combination therapy at discharge (OR, 0.7 [95% CI, 0.6–0.9]), while being FH significantly increased this probability (OR, 1.8 [95% CI, 1.3–2.4]). The probability of having a combination therapy increased significantly between discharge and 1 year, with no difference between FH and non-FH (non-FH: OR, 2.5 [95% CI, 2.0–3.2]; FH: OR, 2.9 [95% CI, 2.0–4.1]; *P* value of difference=0.587). The increase was also significant between 1 and 5 years, and it was significantly greater for FH at 10% significance level (no FH: OR, 3.8 [95% CI, 3.1–4.6]; FH: OR, 5.1 [95% CI, 4.0–6.7]; *P* value of the difference=0.063). No interaction between sex and time or between sex and FH was found to be significant, thus it was not retained in the model.

**Figure 2.**
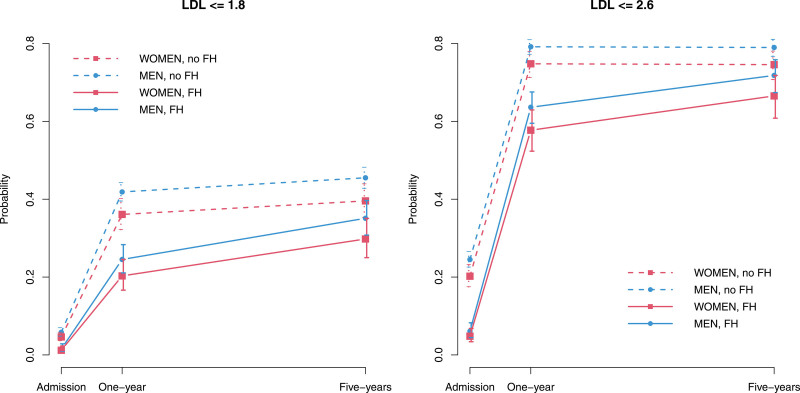
Progression of achievement of target LDL-c values from hospital admission to 5 years post-ACS for males and females with and without FH* estimated via generalized estimating equation models (n=3139). *All definitions of FH used (Dutch Lipid Clinic Network possible and probable FH and Simon Broome register). ACS indicates acute coronary syndrome; FH, familial hypercholesterolemia; LDL-c, low-density lipoprotein-cholesterol, and LLT, lipid-lowering therapy.

Regarding LDL-c goal achievement (Figure 2; Table S8), being female corresponded to a lower probability of having LDL-c ≤1.8 mmol/L at hospital admission (OR, 0.8 [95% CI, 0.7–0.9]), and being FH also significantly reduced this probability (OR, 0.5 [95% CI, 0.4–0.6]), according to our generalized estimating equation models. The probability of LDL-c ≤1.8 mmol/L increased significantly between hospital admission and 1 year and between 1 and 5 years. The increase was significantly greater for FH at both 1 year (non-FH: OR, 11.7 [95% CI, 9.6–14.2]; FH: OR, 21.1 [95% CI, 11.2–39.6]; *P* value of difference=0.080) and between 1 and 5 years (non-FH: OR, 1.2 [95% CI, 1.0–1.3]; FH: OR, 1.7 [95% CI, 1.3–2.1]; *P* value of difference=0.008). No interaction between sex and time or between sex and FH was found to be significant (thus not included in the model). Similar results were found for the target LDL-c ≤2.6 mmol/L.

All models were also estimated, adjusting for age, level of education, and study site. No relevant differences were found compared with unadjusted models, with the exception of the effect of sex on the probability of having combination therapy at discharge, which became weaker (OR, 0.9 [95% CI, 0.7–1.1]; data not shown).

## DISCUSSION

In this large prospective cohort study that included over 600 females with CAD and over 700 patients with clinical FH with a follow-up of 5 years after ACS, we found that there were large sex disparities in LLT use, with females having less optimal lipid-lowering treatment than males, independently of the presence of FH. This included the probability of having no statin and of less frequently having a combination LLT. Furthermore, females were less likely to reach target LDL-c values at the 5-year follow-up as compared with males, independent of FH status. Second, target LDL-c levels were less frequently reached in patients with FH than in those without FH, despite higher use of high-dose statins or combination LLT 5 years after ACS. Moreover, only half of the patients with FH were still on high-dose statins as prescribed at hospital discharge, and only one third were on a combination therapy 5 years post-ACS.

Our results about sex differences are similar to another study in the general population, showing that females are less likely than males to be taking statins 1 year after ACS.^27^ Here, we further report that this difference persists in the long term after ACS. A study by Perez de Isla et al^9^ with a 5-year follow-up of patients with FH in the Spanish SAFEHEART Registry (Spanish Familial Hypercholesterolemia Cohort Study) reported that females with FH have a lower OR than males of using high-dose statins, but this result is not stratified by the presence of ASCVD. We also found that females had a higher probability of not having any LLT at all at 5 years post-ACS as compared with males and that they were less likely than males to be receiving combination therapy. These differences in management may be particularly problematic for females with FH because they have a life-long cardiovascular risk similar to males at a young age.^28^ One of the explanations for females having a lower use of statins is a higher prevalence of side effects from statins.^27,29,30^ One study found that other factors that play a role in lower prescription rates of statins for females after ACS are females’ higher age at the time of ACS than males, their lower cardiovascular risk due to a lower prevalence of smoking in females as compared with males, and females having less cardiologist evaluations.^27^ Nevertheless, the 2016 and 2019 ESC guidelines report that both sexes derive the same benefit from LLT; therefore, more attention is needed to increase the quality of care and prevention in terms of LLT in females after ACS.

We also reported the differences in LLT use between patients with and without FH. The difficulty for patients with FH to maintain recommended target LDL-c values over a long period of time after an ACS has been poorly studied. Previous cross-sectional studies have investigated LDL-c attainment in FH populations, but the patients included are in both primary and secondary prevention, and the time after ACS is not specified.^31,32^ Another study by Iyen et al^5^ included only patients in primary prevention, and follow-up was shorter, at 12 and 24 months after inclusion. The European Atherosclerosis Society FH Studies Collaboration had a large study sample of roughly 11 000 patients with FH in secondary prevention but reported cross-sectional data only with no indication of when ACS took place.^3^ Only the study by Perez de Isla et al^9^ had a mean 5-year follow-up of patients with FH. Their study sample included patients with and without ASCVD and found that the presence of previous ASCVD was independently associated with difficulty in attaining target LDL-c levels, suggesting that clinically significant LDL-c burden is more difficult to reverse. Our study reinforces this message based on a larger study sample of patients with FH in secondary prevention. Similar results have been found in the general population in the DA VINCI cohort,^33,34^ where patients in primary prevention reach their respective LDL-c targets more frequently than patients in secondary prevention. A possible explanation for patients with FH in secondary prevention having more intensive treatment is a higher risk perceived by physicians due to the higher prevalence of a positive family and personal history of premature ASCVD and higher untreated LDL-c values in FH. The resistance of patients with FH to reach target LDL-c values probably stems from the nature of their genetic disease, with a constant over-production of LDL-c despite LLT use.

Recent data in the international SANTORINI study (Treatment of High and Very High Risk Dyslipidemic Patients for the Prevention of Cardiovascular Events in Europe–A Multinational Observational Study)^33^ by Ray et al reported that at 1-year follow-up, combination therapy was used in 26.4% of patients in the very high-risk group, 7.5% of whom had FH. In our study sample composed only of very high-risk patients with 23.5% of patients with FH, the use of combination LLT in patients with FH and non-FH combined was slightly lower at 21.5%. Hopefully, the prescription of combination LLT will continue to rise for patients in secondary prevention, bringing them closer to reaching target LDL-c values.

Our study has several limitations. First, the definition of FH was based on clinical variables without genetic tests being performed. This could lead to a potential misclassification of monogenic forms of FH. However, the standard of care is largely based on the clinical definition of FH in Switzerland, as genetic tests are not reimbursed by health insurances. Second, at 1- and 5-year follow-up visits, information was based on patient knowledge; therefore, patients who were not properly informed on their medication or the dosage of their treatment could have been misclassified. Furthermore, our data on PCSK9 inhibitors is very limited, as only a small percentage of patients were taking them at the 5-year follow-up visit. Finally, as reported in Figure S1, not all patients in the cohort had a 5-year follow-up visit, explaining the large number of missing values at the follow-up time points. Our study sample was based on centers and not on patient characteristics, without sex differences between patients who did and did not participate, as reported in Table S1. Therefore, we believe that our study sample at 5 years had sufficient external validity and was larger than in previous studies.

## CONCLUSIONS

Five years after ACS, LLT use and target LDL-c achievement for females were systematically worse in the long term after ACS compared with men. Furthermore, the lipid management of patients with FH was insufficient. With the large panel of lipid-lowering drugs currently available, more stringent management, such as an increase in the prescription of combination LLT, could lead to higher rates of LDL-c target attainment and reduced recurrent cardiovascular events in males and females, both with and without FH, after ACS.

## ARTICLE INFORMATION

### Acknowledgments

The authors acknowledge the cooperation of all participating centers, practicing physicians, referring doctors and institutions.

### Sources of Funding

The SPUM-ACS (Special Program University Medicine-Acute Coronary Syndrome) cohort was supported by the Swiss National Science Foundation (SNSF 33CM30-124112, Inflammation and acute coronary syndromes (ACS) – Novel strategies for prevention and clinical management; and SNSF 32473B_163271, Long-term benefit of the multi-center, multi-dimensional secondary prevention program in patients with acute coronary syndromes). None of the funding bodies had any role in design and conduct of the study; collection, management, analysis, and interpretation of the data; and preparation, review, or approval of the manuscript.

### Disclosures

Dr Matter reports receiving grants from Merck Sharp & Dohme, Eli Lilly, AstraZeneca, Roche, Bayer, Novo Nordisk; expert testimony from Merck Sharp & Dohme; payment for lectures from Merck Sharp & Dohme, AstraZeneca, and Roche. Dr Mach has received honoraria for advisory boards and conferences on dyslipidemia from Amgen, AstraZeneca, Bristol Myers Squibb, Eli Lilly, Merck Sharp & Dohme, Sanofi, and Pfizer. The other authors report no conflicts.

### Supplemental Material

Figures S1–S4

Tables S1–S8

## Supplementary Material



## References

[1] Shah T Haimi I Yang Y Gaston S Taoutel R Mehta S Jae Lee H Zambahari R Baumbach A Henry TD. Meta-analysis of gender disparities in in-hospital care and outcomes in patients with ST-segment elevation myocardial infarction. Am J Cardiol. 2021;147:23–32. doi: 10.1016/j.amjcard.2021.02.015 33640366 10.1016/j.amjcard.2021.02.015

[2] Zhao M Woodward M Vaartjes I Millett ERC Klipstein-Grobusch K Hyun K Carcel C Peters SAE. Sex differences in cardiovascular medication prescription in primary care: a systematic review and meta-analysis. J Am Heart Assoc. 2020;9:e014742. doi: 10.1161/JAHA.119.014742 32431190 10.1161/JAHA.119.014742 PMC7429003

[3] Vallejo-Vaz AJ Stevens CAT Lyons ARM Dharmayat KI Freiberger T Hovingh GK Mata P Raal FJ Santos RD Soran H. Global perspective of familial hypercholesterolaemia: a cross-sectional study from the EAS Familial Hypercholesterolaemia Studies Collaboration (FHSC). Lancet. 2021;398:1713–1725. doi: 10.1016/S0140-6736(21)01122-3 34506743 10.1016/S0140-6736(21)01122-3

[4] Mach F Baigent C Catapano AL Koskinas KC Casula M Badimon L Chapman MJ De Backer GG Delgado V Ference BA. 2019 ESC/EAS guidelines for the management of dyslipidaemias: lipid modification to reduce cardiovascular risk. Eur Heart J. 2020;41:111–188. doi: 10.1093/eurheartj/ehz455 31504418 10.1093/eurheartj/ehz455

[5] Iyen B Akyea RK Weng S Kai J Qureshi N. Statin treatment and LDL-cholesterol treatment goal attainment among individuals with familial hypercholesterolaemia in primary care. Open Heart. 2021;8:e001817. doi: 10.1136/openhrt-2021-001817 34702779 10.1136/openhrt-2021-001817 PMC8549660

[6] Bérard E Bongard V Haas B Dallongeville J Moitry M Cottel D Ruidavets JB Ferrières J. Prevalence and treatment of familial hypercholesterolemia in France. Can J Cardiol. 2019;35:744–752. doi: 10.1016/j.cjca.2019.02.013 31151710 10.1016/j.cjca.2019.02.013

[7] Brunham LR Cermakova L Lee T Priecelova I Alloul K De Chantal M Francis GA Frohlich J. Contemporary trends in the management and outcomes of patients with familial hypercholesterolemia in Canada: a prospective observational study. Can J Cardiol. 2017;33:385–392. doi: 10.1016/j.cjca.2016.08.016 27931859 10.1016/j.cjca.2016.08.016

[8] deGoma EM Ahmad ZS O’Brien EC Kindt I Shrader P Newman CB Pokharel Y Baum SJ Hemphill LC Hudgins LC. Treatment gaps in adults with heterozygous familial hypercholesterolemia in the United States: data from the CASCADE-FH registry. Circ Cardiovasc Genet. 2016;9:240–249. doi: 10.1161/CIRCGENETICS.116.001381 27013694 10.1161/CIRCGENETICS.116.001381 PMC5315030

[9] Perez De Isla L Alonso R Watts GF Mata N Saltijeral Cerezo A Muñiz O Fuentes F Diaz-Diaz JL de Andres R Zambon D. Attainment of LDL-cholesterol treatment goals in patients with familial hypercholesterolemia. J Am Coll Cardiol. 2016;67:1278–1285. doi: 10.1016/j.jacc.2016.01.008 26988947 10.1016/j.jacc.2016.01.008

[10] Schreuder MM Hamkour S Siegers KE Holven KB Johansen AK Van De Ree MA Imholz B Boersma E Louters L Bogsrud MP. LDL cholesterol targets rarely achieved in familial hypercholesterolemia patients: a sex and gender-specific analysis. Atherosclerosis. 2023;384:117117. doi: 10.1016/j.atherosclerosis.2023.03.022 37080805 10.1016/j.atherosclerosis.2023.03.022

[11] De Luca L Arca M Temporelli PL Colivicchi F Gonzini L Lucci D Bosco B Callerame M Lettica GV Di Lenarda A. Prevalence and pharmacologic management of familial hypercholesterolemia in an unselected contemporary cohort of patients with stable coronary artery disease. Clin Cardiol. 2018;41:1075–1083. doi: 10.1002/clc.23031 30039543 10.1002/clc.23031 PMC6489755

[12] Lorca R Aparicio A Cuesta-Llavona E Pascual I Junco A Hevia S Villazon F Hernandez-Vaquero D Rodriguez Reguero JJ Moris C. Familial hypercholesterolemia in premature acute coronary syndrome. insights from CholeSTEMI registry. J Clin Med. 2020;9:3489. doi: 10.3390/jcm9113489 33137929 10.3390/jcm9113489 PMC7692119

[13] Arora S Stouffer GA Kucharska-Newton AM Qamar A Vaduganathan M Pandey A Porterfield D Blankstein R Rosamond WD Bhatt DL. Twenty year trends and sex differences in young adults hospitalized with acute myocardial infarction: the ARIC community surveillance study. Circulation. 2019;139:1047–1056. doi: 10.1161/CIRCULATIONAHA.118.037137 30586725 10.1161/CIRCULATIONAHA.118.037137 PMC6380926

[14] Xia S Du X Guo L Du J Arnott C Lam CSP Huffman MD Arima H Zuan Z Yheng Z. Sex differences in primary and secondary prevention of cardiovascular disease in China. Circulation. 2020;141:530–539. doi: 10.1161/CIRCULATIONAHA.119.043731 32065775 10.1161/CIRCULATIONAHA.119.043731

[15] De Backer G Jankowski P Kotseva K Mirrakhimov E Reiner Z Rydén L Tokgözoglu L Wood D De Bacquier D. Management of dyslipidaemia in patients with coronary heart disease: results from the ESC-EORP EUROASPIRE V survey in 27 countries. Atherosclerosis. 2019;285:135–146. doi: 10.1016/j.atherosclerosis.2019.03.014 31054483 10.1016/j.atherosclerosis.2019.03.014

[16] Ryzhaya N Cermakova L Trinder M Ruel I Coutinho T Genest J Brunham LR. Sex differences in the presentation, treatment, and outcome of patients with familial hypercholesterolemia. J Am Heart Assoc. 2021;10:e019286. doi: 10.1161/JAHA.120.019286 34032141 10.1161/JAHA.120.019286 PMC8483526

[17] Nanchen D Gencer B Auer R Räber L Stefanini GG Klingenberg R Schmied CM Cornuz J Muller O Vogt P. Prevalence and management of familial hypercholesterolaemia in patients with acute coronary syndromes. Eur Heart J. 2015;36:2438–2445. doi: 10.1093/eurheartj/ehv289 26142466 10.1093/eurheartj/ehv289

[18] Auckle R Su B Li H Xu S Xie M Song Y Quddus MA Xu Y Liu B Che W. Familial hypercholesterolemia in Chinese patients with premature ST-segment-elevation myocardial infarction: prevalence, lipid management and 1-year follow-up. PLoS One. 2017;12:e0186815. doi: 10.1371/journal.pone.0186815 29088271 10.1371/journal.pone.0186815 PMC5663389

[19] Singh A Gupta A Collins BL Qamar A Monda KL Biery D Lopez JAG de Ferranti SD Plutzky J Cannon CP. Familial hypercholesterolemia among young adults with myocardial infarction. J Am Coll Cardiol. 2019;73:2439–2450. doi: 10.1016/j.jacc.2019.02.059 31097165 10.1016/j.jacc.2019.02.059

[20] Masana L Zamora A Plana N Comas-Cufí M Garcia-Gil M Martí-Lluch R Ponjoan A Alves-Cabratosa L Elosua R Marrugat J. Incidence of cardiovascular disease in patients with familial hypercholesterolemia phenotype: analysis of 5 years follow-up of real-world data from more than 1.5 million patients. J Clin Med. 2019;8:1080. doi: 10.3390/jcm8071080 31340450 10.3390/jcm8071080 PMC6678686

[21] Nanchen D Gencer B Muller O Auer R Aghlmandi S Heg D Klingenberg R Räber L Carballo D Carballo S. Prognosis of patients with familial hypercholesterolemia after acute coronary syndromes. Circulation. 2016;134:698–709. doi: 10.1161/CIRCULATIONAHA.116.023007 27462068 10.1161/CIRCULATIONAHA.116.023007

[22] Besseling J Kindt I Hof M Kastelein JJP Hutten BA Hovingh GK. Severe heterozygous familial hypercholesterolemia and risk for cardiovascular disease: a study of a cohort of 14,000 mutation carriers. Atherosclerosis. 2014;233:219–223. doi: 10.1016/j.atherosclerosis.2013.12.020 24529147 10.1016/j.atherosclerosis.2013.12.020

[23] Visseren FLJ Mach F Smulders YM Carballo D Koskinas KC Bäck M Benetos A Biffi A Boavida JM Capodanno D; ESC National Cardiac Societies. 2021 ESC guidelines on cardiovascular disease prevention in clinical practice. Eur Heart J. 2021;42:3227–3337. doi: 10.1093/eurheartj/ehab484 34458905 10.1093/eurheartj/ehab484

[24] Gidding SS Champagne MA De Ferranti SD Defesche J Ito MK Knowles JW McCrindle B Raal F Rader D Santos RD. The agenda for familial hypercholesterolemia: a scientific statement from the American Heart Association. Circulation. 2015;132:2167–2192. doi: 10.1161/CIR.0000000000000297 26510694 10.1161/CIR.0000000000000297

[25] Hu P Dharmayat KI Stevens CAT Sharabiani MTA Jones RS Watts GF Genest J Ray KK Vallejo-Vaz AJ. Prevalence of familial hypercholesterolemia among the general population and patients with atherosclerotic cardiovascular disease: a systematic review and meta-analysis. Circulation. 2020;141:1742–1759. doi: 10.1161/CIRCULATIONAHA.119.044795 32468833 10.1161/CIRCULATIONAHA.119.044795

[26] Catapano AL Graham I De Backer G Wiklund O Chapman MJ Drexel H Hoes AW Jennings CS Landmesser U Pedersen TR. 2016 ESC/EAS guidelines for the management of dyslipidaemias. Eur Heart J. 2016;37:2999–3058. doi: 10.1093/eurheartj/ehw272 27567407 10.1093/eurheartj/ehw272

[27] Zhang H Plutzky J Shubina M Turchin A. Drivers of the sex disparity in statin therapy in patients with coronary artery disease: a cohort study. PLoS One. 2016;11:e0155228. doi: 10.1371/journal.pone.0155228 27148965 10.1371/journal.pone.0155228 PMC4858152

[28] Perak AM Ning H De Ferranti SD Gooding HC Wilkins JT Lloyd-Jones DM. Long-term risk of atherosclerotic cardiovascular disease in US adults with the familial hypercholesterolemia phenotype. Circulation. 2016;134:9–19. doi: 10.1161/CIRCULATIONAHA.116.022335 27358432 10.1161/CIRCULATIONAHA.116.022335 PMC4933328

[29] Bots SH Inia JA Peters SAE. Medication adherence after acute coronary syndrome in women compared with men: a systematic review and meta-analysis. Front Glob Womens Health. 2021;2:637398. doi: 10.3389/fgwh.2021.637398 34816194 10.3389/fgwh.2021.637398 PMC8594018

[30] Colvin CL Poudel B Bress AP Derington CG King JB Wen Y Chen L Bittner V Brown TM Monda KL. Race/ethnic and sex differences in the initiation of non-statin lipid-lowering medication following myocardial infarction. J Clin Lipidol. 2021;15:665–673. doi: 10.1016/j.jacl.2021.08.001 34452823 10.1016/j.jacl.2021.08.001

[31] Schmidt N Dressel A Grammer TB Gouni-Berthold I Julius U Kassner U Klose G König C Koenig W Otte B. Lipid-modifying therapy and low-density lipoprotein cholesterol goal attainment in patients with familial hypercholesterolemia in Germany: the CaReHigh registry. Atherosclerosis. 2018;277:314–322. doi: 10.1016/j.atherosclerosis.2018.08.050 30270065 10.1016/j.atherosclerosis.2018.08.050

[32] Vrablik M Raslová K Vohnout B Blaha V Satny M Kyselak O Vaclova M Urbanek R Maskova J Soska V. Real-life LDL-C treatment goals achievement in patients with heterozygous familial hypercholesterolemia in the Czech Republic and Slovakia: Results of the PLANET registry. Atherosclerosis. 2018;277:355–361. doi: 10.1016/j.atherosclerosis.2018.08.008 30270071 10.1016/j.atherosclerosis.2018.08.008

[33] Ray KK Molemans B Schoonen WM Giovas P Bray S Kiru G Murphy J Banach M De Servi S Gaita D. EU-Wide cross-sectional observational study of lipid-modifying therapy use in secondary and primary care: the DA VINCI study. Eur J Prev Cardiol. 2021;28:1279–1289. doi: 10.1093/eurjpc/zwaa047 33580789 10.1093/eurjpc/zwaa047

[34] Van De Borne P Peeters A Janssens L Leone A Lemmens R Verhaegen A De Meulemeester M Balthazar Y Heijmans S Calozet Y. Lipid-lowering therapy and risk-based LDL-C goal attainment in Belgium: DA VINCI observational study. Acta Cardiol. 2022;79:20–29. doi: 10.1080/00015385.2022.2030568 35442151 10.1080/00015385.2022.2030568

