# Supplementary material for: Association Between Patient Sex and Familial Hypercholesterolemia and Long-Term Cardiovascular Risk Factor Management 5 Years After Acute Coronary Syndrome
Source: Circ Cardiovasc Qual Outcomes. 2024 Jun 20;17(8):e010790. doi: 10.1161/CIRCOUTCOMES.123.010790 (PMC11338030; doi:10.1161/CIRCOUTCOMES.123.010790)
Supplement: Supplementary file 1 [file hcq-17-e010790-s001.pdf]

SUPPLEMENTAL MATERIAL: Association between patient sex and familial hypercholesterolemia and long-term cardiovascular risk factor management five years after acute coronary syndrome

Kristina Krasieva, MD[1*], Baris Gencer, MD[2,3,4*], Isabella Locatelli, PhD[1], David Carballo, MD[2], Olivier Muller, MD/PhD[4], Stéphane Fournier, MD/PhD[4], Christian M. Matter, MD[5], Lorenz Räber, MD/PhD[6], Nicolas Rodondi, MD[3,7], François Mach, MD[2‡], David Nanchen, MD[1‡]

[1]Center for Primary Care and Public Health (Unisanté), University of Lausanne, Lausanne, Switzerland

[2]Service of Cardiology, Geneva University Hospital, Geneva, Switzerland

[3]Institute of Primary Health Care (BIHAM), University of Bern, Switzerland

[4]Service of Cardiology, Lausanne University Hospital, Lausanne, Switzerland

[5]University Heart Center, Department of Cardiology, Zurich University Hospital, and Center of Translational and Experimental Cardiology (CTEC), Department of Cardiology, Zurich University Hospital and University of Zurich, Zurich, Switzerland

[6]Department of Cardiology, Inselspital, Bern University Hospital, University of Bern, Bern, Switzerland

[7]Department of General Internal Medicine, Inselspital, Bern University Hospital, Bern, Switzerland

[*]both first authors have equal authorship

[‡]both last authors have equal authorship

Corresponding author: David Nanchen, MD, MSc, Center for Primary Care and Public Health (Unisanté), University of Lausanne, Rue du Bugnon 44, CH-1011 Lausanne, Switzerland, Tel: + 41 21 314 60 53, Fax: +41 21 314 61 08, E-mail: david.nanchen@unisante.ch

Figure S1 – Flow chart of study participants

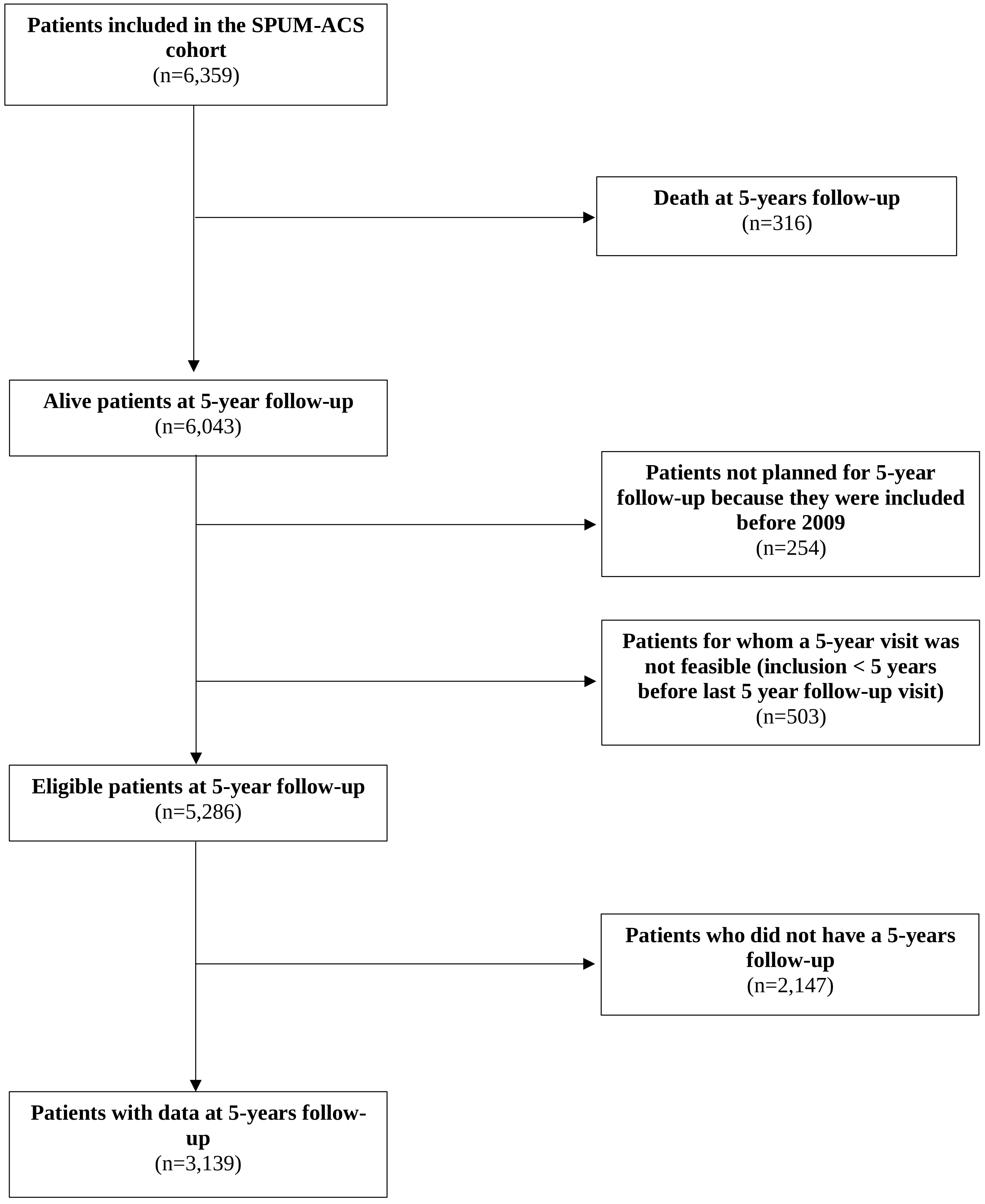

Abbreviations: SPUM-ACS, special program university medicine – acute coronary syndrome

Figure S2 – Cardiovascular risk factor management in patients with and without FH 5 years after ACS

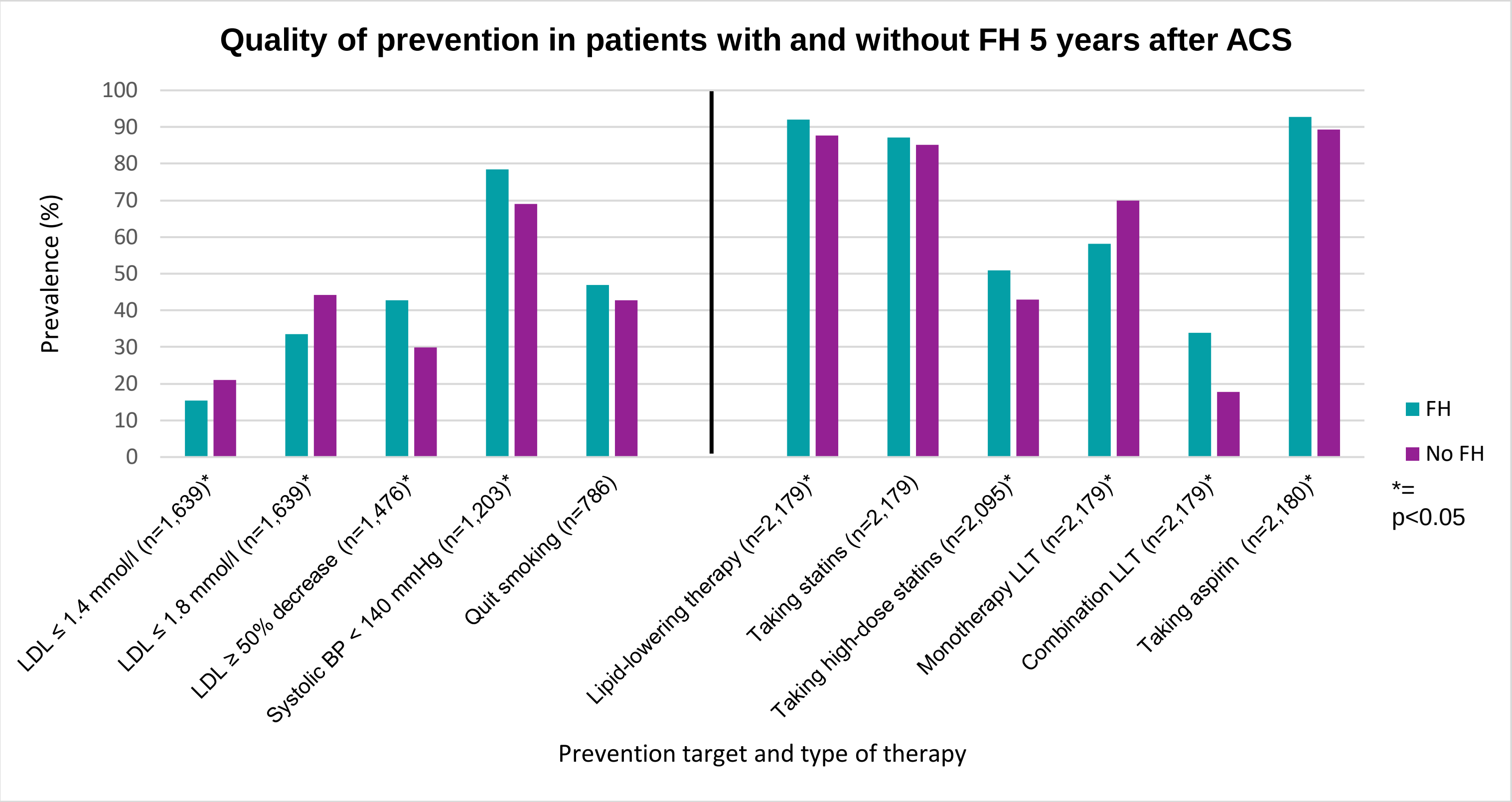

Abbreviations: FH, familial hypercholesterolemia; ACS, acute coronary syndrome; LDL, low-density lipoprotein-cholesterol; BP, blood pressure; LLT, lipid-lowering therapy

Figure S3 – Type of lipid-lowering therapy (LLT*) in patients with † and without FH, 1 year and 5 years after ACS

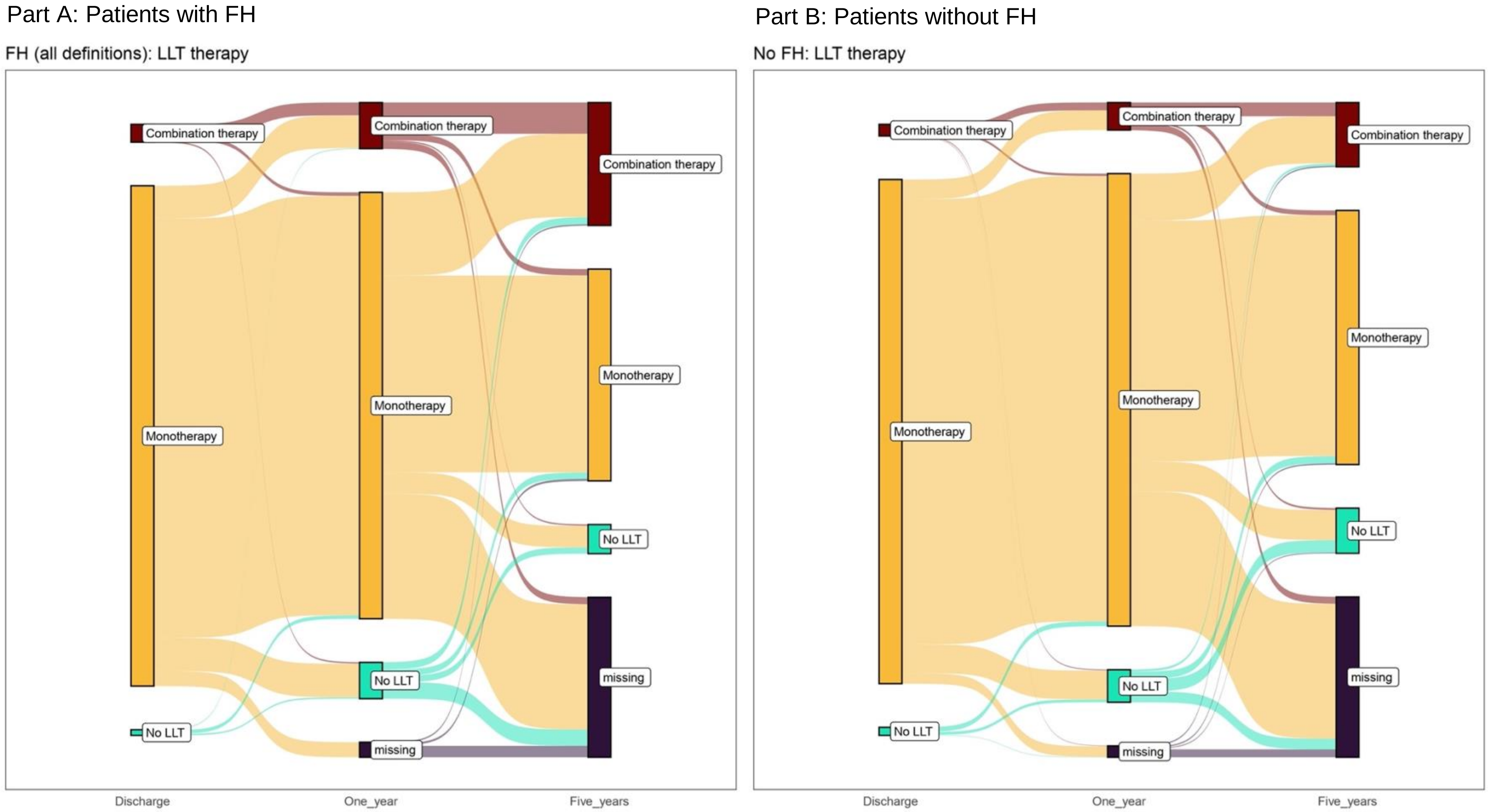

*Use of statins, ezetimibe, fibrates or niacin, or PCSK9 inhibitors (at 5 years only)
† All definitions of FH used (DLCN possible and probable FH and Simon Broome register)
Monotherapy: defined as use of only one of the LLTs
Combination therapy: defined as:

- Discharge and 1 year: a combination of statin + ezetimibe, statin+ niacin, statin + fibrates, niacin + ezetimibe
- 5 years: all of the above and statin + PCSK9 inhibitors +/- ezetimibe, PCSK9 inhibitors + ezetimibe, PCSK9 inhibitors + fibrates, PCSK9 inhibitors + niacin

Abbreviations: FH, familial hypercholesterolemia; ACS, acute coronary syndrome

Figure S4 – Statin dose intensity of patients on statin treatment with* and without FH at discharge, 1 year and 5 years after ACS

Part A: Patients with FH

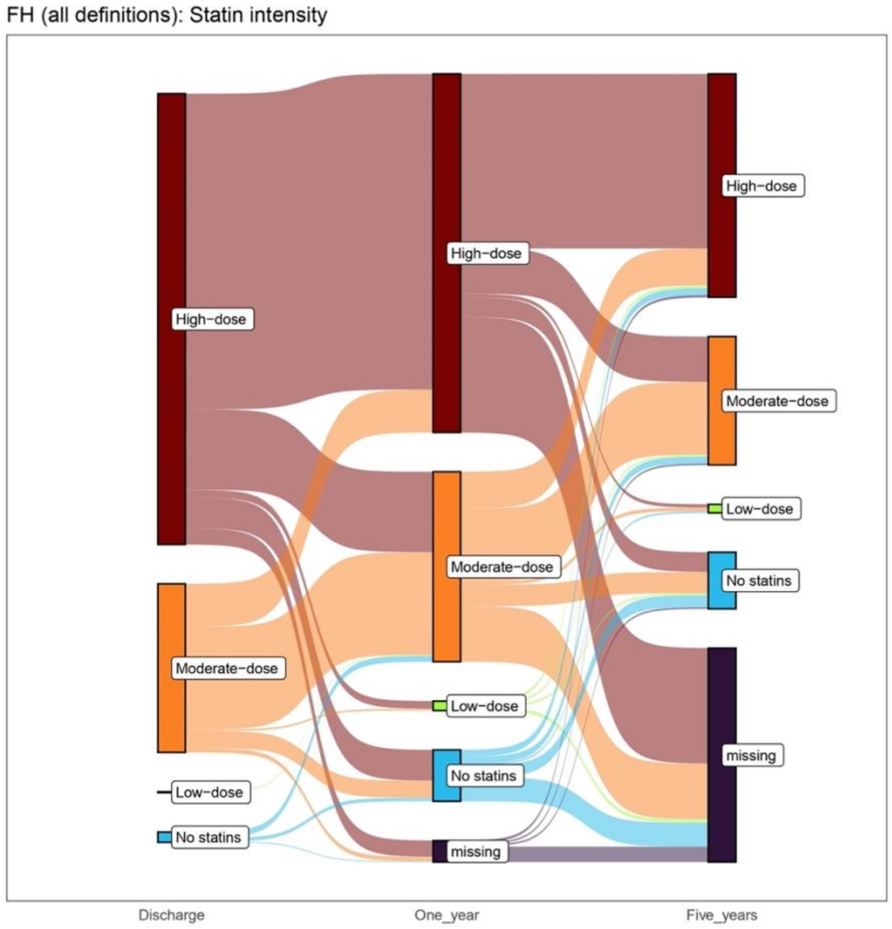

Part B: Patients without FH

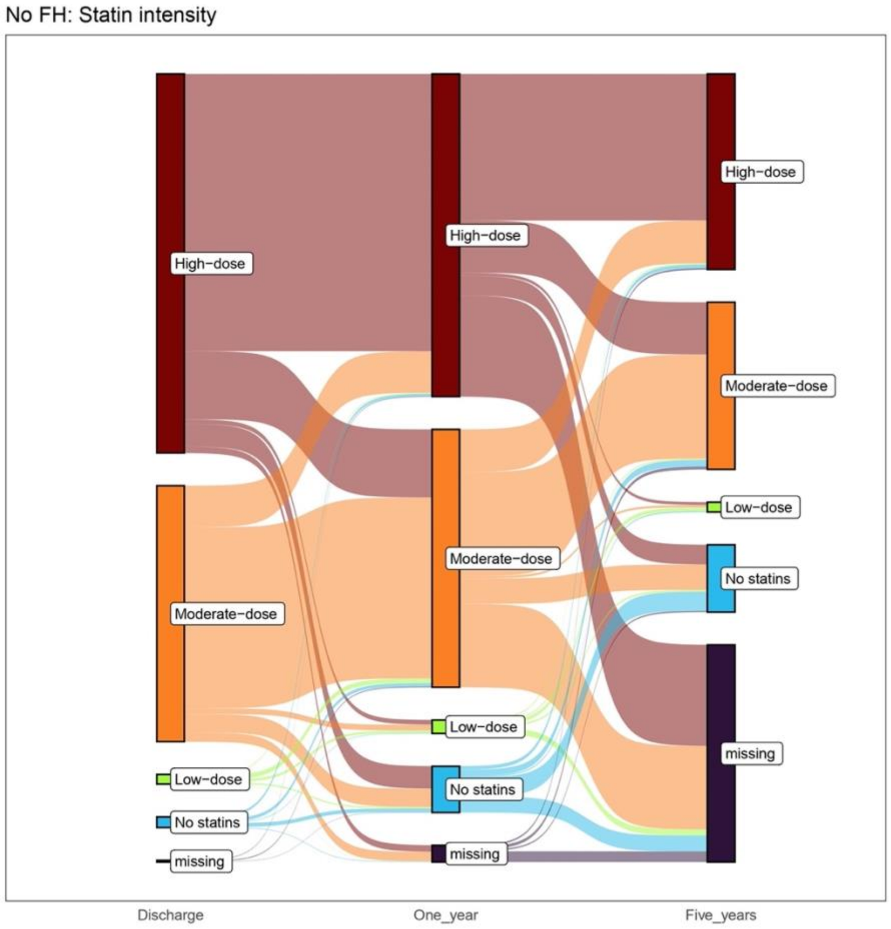

*All definitions of FH used (DLCN possible and probable FH, Simon Broome register)
High-dose statin defined as atorvastatin ≥ 40 mg/d or rosuvastatin ≥ 20 mg/d
Abbreviations: FH, familial hypercholesterolemia; ACS, acute coronary syndrome

Table S1 – Comparison of baseline characteristics of patients who had and didn't have a 5-year follow-up visit

**Demographics**	**Had a 5-year follow-up** (n=3,139)	**Didn't have a 5-year follow-up** (n=2,147)	**p-value**
Age, years	61.4	63.9	<0.001
Female	620 (19.8)	453 (21.1)	0.23
Higher education* (n=4,844)	1,024 (33.5)	452 (25.3)	<0.001
Familial hypercholesterolemia †	659 (21.0)	362 (16.9)	<0.001

Data are given as mean (standard deviation) or number (percentage), unless indicated
*Defined as a high school or university graduation or higher
† Defined as a Dutch Lipid Clinic Network score ≥ 3 points or a "possible FH" based on the Simon Broome criteria

Table S2 – Baseline characteristics of males and females stratified by FH status (n=3,139)

	No FH (n=2,402)	All FH definitions (n=737)	P-value	Possible FH (DLCN 3-5) (n=662)	Probable or definite FH (DLCN > 5) (n=68)	Simon Broome possible FH (n=83)
**Demographics**						
Age, years	64.4 (11.1)	51.6 (9.7)	<0.001	51.7 (9.5)	48.5 (9.4)	54.1 (11.9)
Female	473 (19.7)	147 (20.0)	0.89	130 (19.6)	14 (20.6)	24 (28.9)
Higher education* (n=3,058)	756 (32.4)	268 (37.1)	0.02	244 (37.7)	20 (29.4)	31 (37.8)
Married	1,519 (63.2)	481 (65.3)	0.32	432 (65.3)	45 (66.2)	46(55.4)
Living alone (n=3,122)	592 (24.8)	163 (22.3)	0.18	151 (23.1)	9 (13.2)	21 (25.6)
**Habits**						
Alcohol consumption, units/week (n=2,993)	7.5 (10.0)	6.8 (9.5)	0.10	6.7 (9.4)	7.6 (10.3)	6.9 (9.3)
At-risk alcohol use † (n=3,082)	425 (18.0)	117 (16.1)	0.23	103 (15.8)	12 (17.9)	14 (17.1)
Current smoking (n=3,139)	876 (36.5)	433 (58.8)	<0.001	391 (59.1)	42 (61.8)	45 (54.2)
**Comorbidities**						
Hypertension ‡ (n=3,138)	1,354 (56.4)	280 (38.0)	<0.001	251 (37.9)	25 (36.8)	38 (45.8)
Diabetes mellitus ‖ (n=3,138)	425 (17.7)	79 (10.7)	<0.001	72 (10.9)	6 (8.8)	7 (8.4)
LDL-cholesterol in mmol/l (n=2,836)	2.93 (0.91)	4.19 (1.21)	<0.001	4.02 (1.08)	5.73 (1.28)	5.16 (1.13)
Pre-existing CVD (n=3,137)	584 (24.3)	112 (15.2)	<0.001	99 (15.0)	11 (16.2)	11 (13.3)
Obesity # (n=3,139)	485 (20.2)	179 (24.3)	0.02	157 (23.7)	21 (30.9)	23 (27.7)
Family history of premature CAD** (n=3,124)	443 (18.6)	416 (56.5)	<0.001	359 (54.3)	50 (73.5)	83 (100.0)

**Medication use at admission**						
Lipid-lowering therapy †† (n=1,941)	693 (43.5)	171 (49.4)	0.04	144 (46.6)	25 (71.4)	30 (68.2)
Monotherapy (n=1,941)	636 (39.9)	161 (46.5)	0.07	135 (43.7)	25 (71.4)	27 (61.4)
Statin alone (n=838)	611 (90.9)	150 (90.9)	0.24	127 (91.4)	22 (91.7)	24 (82.8)
Ezetimibe alone (n=838)	9 (1.3)	5 (3.0)	0.24	3 (2.2)	2 (8.3)	2 (6.9)
Combination therapy ‡‡ (n=1,941)	57 (3.6)	10 (2.9)	0.07	9 (2.9)	0 (0.0)	3 (6.8)
Statin + ezetimibe (n=838)	53 (7.9)	10 (6.1)	0.24	9 (6.5)	0 (0.0)	3 (10.3)
Statin (n=1,941)	667 (41.8)	160 (46.2)	0.13	136 (44.0)	22 (62.9)	27 (61.4)
High-dose statin |||| (n=1,941)	172 (10.8)	55 (15.9)	0.01	45 (14.6)	10 (28.6)	9 (20.5)
Aspirin (n=1,945)	675 (42.2)	139 (40.2)	0.49	118 (38.2)	19 (54.3)	20 (45.5)
Antidiabetic medication (n=1,944)	304 (19.0)	47 (13.6)	0.02	44 (14.2)	2 (5.7)	4 (9.1)

Data are given as mean (standard deviation) or number (percentage), unless indicated
Abbreviations: FH, familial hypercholesterolemia; LDL-cholesterol, low-density lipoprotein-cholesterol; CVD, cardiovascular disease; CAD, coronary artery disease

*Defined as a high school or university graduation or higher
† Defined as self-reported more than 14 drinks per week
‡ Defined as systolic BP ≥ 140 mmHg and diastolic BP ≥ 90 mmHg
|| Based on as self-reported and medication information
Defined as a body mass index (BMI) ≥ 30 kg/m^2
**Defined as a history of coronary artery disease in first-degree male relatives < 55 years old and first-degree female relatives < 65 years old
†† Defined as use of statins, ezetimibe, fibrates or niacin
‡‡Defined as use of any combination of statins, ezetimibe, fibrates or niacin
|||| Defined as atorvastatin ≥ 40 mg/day or rosuvastatin ≥ 20 mg/day

Table S3 – LDL-cholesterol goal attainment and lipid-lowering therapy in patients 5 years after ACS, with and without FH (all definitions) (n=3,139)

	**No FH (n=2,402)**	**All FH definitions (n=737)**	**P-value**	**Possible FH (DLCN 3-5) (n=662)**	**Probable or definite FH (DLCN > 5) (n=68)**	**Simon Broome Possible FH (n=83)**
**LDL-cholesterol**						
LDL-cholesterol in mmol/l (n=1,639)	2.06 (0.87)	2.31 (1.00)	<0.001	2.26 (0.91)	2.65 (1.47)	2.73 (1.51)
≤ 1.4 mmol/l (n=1,639)	259 (21.0)	62 (15.4)	0.01	54 (15.0)	8 (20.5)	8 (17.8)
≤ 1.8 mmol/l (n=1,639)	547 (44.3)	135 (33.5)	<0.001	124 (34.5)	10 (25.6)	12 (26.7)
≤ 2.6 mmol/l (n=1,639)	965 (78.1)	283 (70.2)	0.001	258 (71.9)	23 (59.0)	26 (57.8)
50% decrease reached (n=1,476)	327 (29.9)	164 (42.8)	<0.001	140 (41.3)	23 (59.0)	24 (53.3)
**Lipid-lowering therapy**						
Lipid-lowering therapy* (n=2,179)	1,460 (87.6)	471 (92.0)	0.006	420 (91.9)	48 (96.0)	58 (93.6)
Statin (n=2,179)	1,421 (85.2)	446 (87.1)	0.29	400 (87.5)	44 (88.0)	51 (82.3)
High-dose statin † (n=2,095)	715 (42.9)	261 (51.0)	0.001	229 (50.1)	31 (62.0)	37 (59.7)
Monotherapy LLT ‡ (n=2,179)	1,165 (69.9)	298 (58.2)	<0.001	275 (60.2)	22 (44.0)	29 (46.8)
Statin alone (n= 2,179)	1,133 (68.0)	278 (54.3)	<0.001	257 (56.2)	21 (42.0)	26 (41.9)
Ezetimibe alone (n= 2,179)	17 (1.0)	12 (2.3)	<0.001	10 (2.2)	1 (2.0)	2 (3.2)
PCSK9 inhibitor alone (n= 2,179)	10 (0.6)	5 (1.0)	<0.001	5 (1.1)	0 (0.0)	1 (1.6)

Combination LLT || (n=2,179)	295 (17.7)	173 (33.8)	<0.001	145 (31.7)	26 (52.0)	29 (46.8)
Statin + ezetimibe (n= 2,179)	279 (16.7)	160 (31.3)	<0.001	138 (30.2)	20 (40.0)	22 (35.5)
Statin + PCSK9 inhibitor (n= 2,179)	3 (0.2)	2 (0.4)	<0.001	1 (0.2)	1 (2.0)	1 (1.6)

Data are given as mean (standard deviation) or number (percentage), unless indicated
Abbreviations: LDL-cholesterol, low-density lipoprotein-cholesterol; ACS, acute coronary syndrome; FH, familial hypercholesterolemia; PCSK9 inhibitor, proprotein convertase subtilisin-kexin type 9 inhibitor

*****Use of statins, ezetimibe, fibrates or niacin, or PCSK9 inhibitors
† Defined as atorvastatin ≥ 40 mg/day or rosuvastatin ≥ 20 mg/day
‡ Defined as use of only one of the LLTs (see below)
|| Defined as a combination of statin + ezetimibe, statin+ niacin, statin + fibrates, or niacin + ezetimibe, statin + PCSK9 inhibitors +/- ezetimibe, PCSK9 inhibitors + ezetimibe, PCSK9 inhibitors + fibrates, PCSK9 inhibitors + niacin

Table S4 – Cardiovascular risk factor management in patients 5 years after ACS, with and without FH (all definitions) (n=3,139)

	No FH (n=2,402)	All FH definitions (n=737)	P-value	Possible FH (DLCN 3-5) (n=662)	Probable or definite FH (DLCN > 5) (n=68)	Simon Broome Possible FH (n=83)
**Blood pressure**						
Mean systolic BP in mmHg (n=1,203)	133.0 (15.7)	130.2 (15.7)	0.006	130.5 (15.8)	125.8 (13.3)	133.2 (16.5)
Mean diastolic BP in mmHg (n=1,203)	78.7 (9.9)	80.9 (9.8)	<0.001	81.1 (9.9)	79.3 (8.9)	81.5 (8.4)
Mean systolic BP < 140 mmHg in those with systolic BP ≥ 140 mmHg at 1-year (n=337)	140 (50.2)	31 (53.5)	0.65	29 (56.9)	2 (40.0)	2 (20.0)
Mean diastolic BP < 90 mmHg in those with diastolic BP ≥ 90 mmHg at 1-year (n=160)	92 (75.4)	24 (63.2)	0.14	19 (59.4)	5 (83.3)	3 (75.0)
**HbA1c**						
Reached HbA1c < 7% if diabetic (n=214)	68 (38.9)	18 (46.2)	0.40	15 (44.1)	3 (75.0)	4 (80.0)
**Other medication**						
Aspirin (n=2,180)	1,490 (89.3)	475 (92.8)	0.02	425 (93.0)	45 (90.0)	54 (87.1)
Antihypertensive medication* (n=2,179)	1,478 (88.7)	422 (82.4)	p<0.001	376 (82.3)	42 (84.0)	53 (85.5)
Preventive drugs (aspirin + statin) (n=2,179)	1,285 (77.1)	417 (81.5)	0.04	374 (81.8)	41 (82.0)	45 (72.6)
**Smoking**						
Quit smoking after ACS (n= 786)	227 (42.7)	119 (46.9)	0.27	102 (45.3)	17 (58.6)	18 (58.1)
**Weight change**						

5% weight loss in overweight or obese (n=945)	190 (26.9)	45 (18.8)	0.01	38 (18.2)	6 (20.7)	6 (21.4)
**Alcohol**						
EtOH consumption < 14 units/week in those with at-risk consumption at baseline † (n=542)	91 (21.4)	33 (28.2)	0.12	27 (26.2)	4 (33.3)	6 (42.9)

Data are given as mean (standard deviation) or number (percentage), unless indicated
*****Angiotensin-converting enzyme inhibitors, angiotensin II receptor inhibitors, beta-blockers, calcium channel blockers, diuretics
† At-risk consumption defined as more than 14 units of alcohol per week.
Abbreviations: ACS, acute coronary syndrome; FH, familial hypercholesterolemia; BP, blood pressure; HbA1c, glycated hemoglobin; EtOH, ethanol

Table S5 – Comparison of LLT use by males and females with* and without FH, 1 year and 5 years post-ACS

		No LLT (%)	Monotherapy (%)	Combination therapy (%)	p-value
**Discharge**	No FH, males (n=1,929)	1.4	96.1	2.5	0.03
	No FH, females (n=473)	2.3	96.8	0.9	
	FH, males (n=590)	0.5	95.9	3.6	0.01
	FH, females (n=147)	3.4	93.9	2.7	
**One year**	No FH, males (n=1,891)	5.9	88.5	5.7	0.08
	No FH, females (n=459)	8.3	87.6	4.1	
	FH, males (n=572)	5.6	85.1	9.3	0.01
	FH, females (n=144)	13.2	78.5	8.3	
**Five years**	No FH, males (n=1,368)	11.6	70.0	18.4	0.06
	No FH, females (n=299)	16.1	69.2	14.7	
	FH, males (n=406)	7.1	57.4	35.5	0.19
	FH, females (n=106)	11.3	61.3	27.4	

*All definitions of FH used (DLCN possible and probable FH and Simon Broome register)
Abbreviations: LLT, lipid-lowering therapy; FH, familial hypercholesterolemia; ACS, acute coronary syndrome

Table S6 – Comparison of statin intensity use in males and females with* and without FH, 1 year and 5 years post-ACS

		No statin (%)	Low-dose statin (%)	Moderate-dose statin (%)	High-dose statin † (%)	Unknown statin dose (%)	p-value
**Discharge**	No FH, males (n=1,929)	1.5	1.7	38.8	57.8	0.3	0.33
	No FH, females (n=473)	2.3	0.6	39.5	57.3	0.2	
	FH, males (n=590)	1.0	0.0	25.3	73.7	0.0	0.002
	FH, females (n=147)	4.1	0.7	32.7	62.6	0.0	
**One year**	No FH, males (n=1,891)	6.6	1.9	40.0	51.1	0.4	0.09
	No FH, females (n=459)	9.6	2.8	40.7	46.6	0.2	
	FH, males (n=572)	6.8	1.9	31.1	59.6	0.5	0.02
	FH, females (n=144)	14.6	0.0	30.6	54.2	0.7	
**Five years**	No FH, males (n=1,368)	14.0	2.0	36.3	44.4	3.4	0.06
	No FH, females (n=299)	18.4	3.0	38.5	36.1	4.0	
	FH, males (n=406)	11.3	1.2	29.3	53.2	4.9	0.03
	FH, females (n=106)	18.9	4.7	29.3	42.5	4.7	

*All definitions of FH used (DLCN possible and probable FH, Simon Broome register)
† Defined as atorvastatin ≥ 40 mg/d or rosuvastatin ≥ 20 mg/d
Abbreviations: FH, familial hypercholesterolemia; ACS, acute coronary syndrome

Table S7 – Results of GEE models for LLT use at discharge, 1-year and 5-years post-ACS, stratified by sex and FH* status (n=3,139)

**No statin**	**OR**	**95%CI**		**p of the difference**
Females vs males all timepoints	1.61	1.28	2.03	
FH vs no FH at baseline	1.18	0.87	1.6	
1-year vs baseline for no FH	4.72	3.39	6.57	0.630
1-year vs baseline for FH	5.58	3.07	10.2	
5-years vs 1-year for no FH	2.35	1.97	2.80	0.096
5-years vs 1-year for FH	1.70	1.21	2.38	
**Combination therapy**	**OR**	**95%CI**		**p of the difference**
Females vs males all timepoints	0.72	0.55	0.93	
FH vs no FH baseline	1.76	1.3	2.4	
1-year vs baseline for no FH	2.51	1.95	3.24	0.587
1-year vs baseline for FH	2.85	1.96	4.13	
5-years vs 1-year for no FH	3.78	3.14	4.56	0.063
5-years vs 1-year for FH	5.14	3.95	6.68	

*All definitions of FH used (DLCN possible and probable FH, Simon Broome register)

Abbreviations: GEE, generalized estimating equation; LLT, lipid-lowering therapy; ACS, acute coronary syndrome; OR, odds ratio; 95%CI, 95% confidence interval; FH, familial hypercholesterolemia

Table S8 – Results of GEE models for LDL-c target achievement at hospital admission, 1-year and 5-years post-ACS, stratified by sex and FH* status (n=3,139)

**LDL-c ≤ 1.8 mmol/l**	**OR**	**95%CI**		**p of the difference**
Females vs males all timepoints	0.78	0.66	0.93	
FH vs no FH at baseline	0.45	0.37	0.57	
1-year vs baseline for no FH	11.7	9.6	14.2	0.080
1-year vs baseline for FH	21.1	11.2	39.6	
5-years vs 1-year for no FH	1.16	1.02	1.32	0.008
5-years vs 1-year for FH	1.67	1.32	2.11	
**LDL-c ≤ 2.6 mmol/l**	**OR**	**95%CI**		**p of the difference**
Females vs males all timepoints	0.78	0.66	0.92	
FH vs no FH baseline	0.46	0.48	0.56	
1-year vs baseline for no FH	11.7	10.2	13.4	<0.001
1-year vs baseline for FH	27.0	19.1	38.2	
5-years vs 1-year for no FH	0.99	0.84	1.16	0.007
5-years vs 1-year for FH	1.45	1.15	1.84	

*All definitions of FH used (DLCN possible and probable FH, Simon Broome register)
Abbreviations: GEE, generalized estimating equation; LDL-c, low-density lipoprotein-cholesterol; ACS, acute coronary syndrome; OR, odds ratio; 95%CI, 95% confidence interval; FH, familial hypercholesterolemia
