# Supplementary material for: Association Between Patient Sex and Familial Hypercholesterolemia and Long-Term Cardiovascular Risk Factor Management 5 Years After Acute Coronary Syndrome
Source: Circ Cardiovasc Qual Outcomes. 2024 Jun 20;17(8):e010790. doi: 10.1161/CIRCOUTCOMES.123.010790 (PMC11338030; doi:10.1161/CIRCOUTCOMES.123.010790)
Supplement: Supplementary file 2 [file hcq-17-e010790-s002.pdf]

STROBE Statement—checklist of items that should be included in reports of observational studies

	Item No	Recommendation
**Title and abstract**	1	(*a*) Indicate the study's design with a commonly used term in the title or the abstract<br>Page 2, line 6-7 (abstract)
		(*b*) Provide in the abstract an informative and balanced summary of what was done and what was found<br>Page 2, Methods section (lines 6-10) and Results section (lines 11-20)
**Introduction**		
Background/rationale	2	Explain the scientific background and rationale for the investigation being reported<br>Page 6 line 1- page 7 line 5 (Introduction)
Objectives	3	State specific objectives, including any prespecified hypotheses<br>Page 7, lines 3-5 (Introduction)
**Methods**		
Study design	4	Present key elements of study design early in the paper<br>Page 7, lines 9-11 (Study design sub-section)
Setting	5	Describe the setting, locations, and relevant dates, including periods of recruitment, exposure, follow-up, and data collection<br>Page 7 lines 9-21 (Study design and Study population sub-sections)
Participants	6	(*a*) *Cohort study*—Give the eligibility criteria, and the sources and methods of selection of participants. Describe methods of follow-up<br>Page 7 lines 9-21 (Study design and Study population sub-sections)<br>Figure S1, Supplemental Material page 2
Variables	7	Clearly define all outcomes, exposures, predictors, potential confounders, and effect modifiers. Give diagnostic criteria, if applicable<br>Page 7, line 24 – page 9 line 8 (Diagnosis of FH, Outcomes, Co-variables sub-sections)
Data sources/ measurement	8*	For each variable of interest, give sources of data and details of methods of assessment (measurement). Describe comparability of assessment methods if there is more than one group<br>Page 7 line 24 – page 8 line 7 (Diagnosis of FH sub-section), Page 8 line 10 – page 9 line 3 (Outcomes sub-section) and page 9 lines 6-8 (Co-variables sub-section)
Bias	9	Describe any efforts to address potential sources of bias<br>Page 15, lines 8-20 (Discussion)
Study size	10	Explain how the study size was arrived at<br>Page 7 lines 9-21(Study design and Study population sub-sections)<br>Figure S1, Supplemental Material page 2<br>Page 10 lines 10-13 (Results)
Quantitative variables	11	Explain how quantitative variables were handled in the analyses. If applicable, describe which groupings were chosen and why<br>Page 8 line 10-15 and page 8 line 20 – page 9 line 3
Statistical methods	12	(*a*) Describe all statistical methods, including those used to control for confounding<br>Page 9 line 11 – page 10 line 2 (Statistical analyses sub-section)
		(*b*) Describe any methods used to examine subgroups and interactions<br>Page 9 lines 15-23 (Statistical analyses sub-section)

		(*c*) Explain how missing data were addressed<br>Page 7 lines 15-17 (Study design sub-section) and page 7 lines 20-21 (Study population sub-section)<br>Figure S1, Supplemental Material page 2
		(*d*) *Cohort study*—If applicable, explain how loss to follow-up was addressed<br>Page 7 lines 15-17 (Study design sub-section) and page 7 lines 20-21 (Study population sub-section)<br>Figure S1, Supplemental Material page 2
		(*e*) Describe any sensitivity analyses<br>Page 12, lines 22-25
**Results**		
Participants	13*	(a) Report numbers of individuals at each stage of study—eg numbers potentially eligible, examined for eligibility, confirmed eligible, included in the study, completing follow-up, and analysed<br>Page 10 lines 10-13 (Results)<br>Figure S1, Supplemental Material page 2
		(b) Give reasons for non-participation at each stage<br>Figure S1, Supplemental Material page 2
		(c) Consider use of a flow diagram<br>Figure S1, Supplemental Material page 2
Descriptive data	14*	(a) Give characteristics of study participants (eg demographic, clinical, social) and information on exposures and potential confounders<br>Page 10, lines 13-23<br>Page 21, Table 1
		(b) Indicate number of participants with missing data for each variable of interest<br>The final number of participants for who data is available "n" is always written in the tables next to the variable. If there is no "n=", it signifies that there are no missing data
		(c) *Cohort study*—Summarise follow-up time (eg, average and total amount)<br>Page 10, lines 12-13 (Results)
Outcome data	15*	*Cohort study*—Report numbers of outcome events or summary measures over time<br>Page 9 line 25 – page 12 line 25 (Results)
Main results	16	(*a*) Give unadjusted estimates and, if applicable, confounder-adjusted estimates and their precision (eg, 95% confidence interval). Make clear which confounders were adjusted for and why they were included<br>Page 11 line 17 – page 12 line 25 (Results)
		(*b*) Report category boundaries when continuous variables were categorized<br>Page 8 lines 9-15 and page 8 line 20 – page 9 line 3 (Methods, Outcomes sub-section)
		(*c*) If relevant, consider translating estimates of relative risk into absolute risk for a meaningful time period<br>N/A
Other analyses	17	Report other analyses done—eg analyses of subgroups and interactions, and sensitivity analyses<br>Page 11 lines 6-15 (Results)
**Discussion**		
Key results	18	Summarise key results with reference to study objectives<br>Page 13 lines 2-11 (Discussion)

Limitations	19	Discuss limitations of the study, taking into account sources of potential bias or imprecision. Discuss both direction and magnitude of any potential bias<br>Page 15 lines 8-20 (Discussion)
Interpretation	20	Give a cautious overall interpretation of results considering objectives, limitations, multiplicity of analyses, results from similar studies, and other relevant evidence<br>Page 13 line 2 – page 15 line 20 (Discussion)
Generalisability	21	Discuss the generalisability (external validity) of the study results<br>Page 15 – lines 17-20 (Discussion)
**Other information**		
Funding	22	Give the source of funding and the role of the funders for the present study and, if applicable, for the original study on which the present article is based<br>Page 16 lines 5-12

*Give information separately for cases and controls in case-control studies and, if applicable, for exposed and unexposed groups in cohort and cross-sectional studies.

Note: An Explanation and Elaboration article discusses each checklist item and gives methodological background and published examples of transparent reporting. The STROBE checklist is best used in conjunction with this article (freely available on the Web sites of PLoS Medicine at http://www.plosmedicine.org/, Annals of Internal Medicine at http://www.annals.org/, and Epidemiology at http://www.epidem.com/). Information on the STROBE Initiative is available at www.strobe-statement.org.
